# Visual vs Fully Automatic Histogram-Based Assessment of Idiopathic Pulmonary Fibrosis (IPF) Progression Using Sequential Multidetector Computed Tomography (MDCT)

**DOI:** 10.1371/journal.pone.0130653

**Published:** 2015-06-25

**Authors:** Davide Colombi, Julien Dinkel, Oliver Weinheimer, Berenike Obermayer, Teodora Buzan, Diana Nabers, Claudia Bauer, Ute Oltmanns, Karin Palmowski, Felix Herth, Hans Ulrich Kauczor, Nicola Sverzellati, Michael Kreuter, Claus Peter Heussel

**Affiliations:** 1 Department of Surgical Sciences, Section of Diagnostic Imaging at University of Parma, Parma, Italy; 2 Translational Lung Research Centre (TLRC), Member of the German Center for Lung Research (DZL), Heidelberg, Germany; 3 Department of Diagnostic and Interventional Radiology, University of Heidelberg, Heidelberg, Germany; 4 Department of Clinical Radiology, University of Munich, Munich, Germany; 5 Department of Pneumology, Iuliu Hatieganu University of Medicine and Pharmacy in Cluj-Napoca, Cluj-Napoca, Romania; 6 Division of Medical and Biological Informatics (E130), German Cancer Research Center (DFKZ), Heidelberg, Germany; 7 Department of Pneumology and respiratory critical care medicine, Center for interstitial and rare lung diseases, Thoraxklinik at University of Heidelberg, Heidelberg, Germany; University of Miami Miller School of Medicine, UNITED STATES

## Abstract

**Objectives:**

To describe changes over time in extent of idiopathic pulmonary fibrosis (IPF) at multidetector computed tomography (MDCT) assessed by semi-quantitative visual scores (VSs) and fully automatic histogram-based quantitative evaluation and to test the relationship between these two methods of quantification.

**Methods:**

Forty IPF patients (median age: 70 y, interquartile: 62-75 years; M:F, 33:7) that underwent 2 MDCT at different time points with a median interval of 13 months (interquartile: 10-17 months) were retrospectively evaluated. In-house software YACTA quantified automatically lung density histogram (10^th^-90^th^ percentile in 5^th^ percentile steps). Longitudinal changes in VSs and in the percentiles of attenuation histogram were obtained in 20 untreated patients and 20 patients treated with pirfenidone. Pearson correlation analysis was used to test the relationship between VSs and selected percentiles.

**Results:**

In follow-up MDCT, visual overall extent of parenchymal abnormalities (OE) increased in median by 5 %/year (interquartile: 0 %/y; +11 %/y). Substantial difference was found between treated and untreated patients in HU changes of the 40th and of the 80th percentiles of density histogram. Correlation analysis between VSs and selected percentiles showed higher correlation between the changes (Δ) in OE and Δ 40^th^ percentile (*r*=0.69; *p*<0.001) as compared to Δ 80^th^ percentile (*r*=0.58; *p*<0.001); closer correlation was found between Δ ground-glass extent and Δ 40^th^ percentile (*r*=0.66, *p*<0.001) as compared to Δ 80^th^ percentile (*r*=0.47, *p*=0.002), while the Δ reticulations correlated better with the Δ 80^th^ percentile (*r*=0.56, *p*<0.001) in comparison to Δ 40^th^ percentile (*r*=0.43, *p*=0.003).

**Conclusions:**

There is a relevant and fully automatically measurable difference at MDCT in VSs and in histogram analysis at one year follow-up of IPF patients, whether treated or untreated: Δ 40^th^ percentile might reflect the change in overall extent of lung abnormalities, notably of ground-glass pattern; furthermore Δ 80^th^ percentile might reveal the course of reticular opacities.

## Introduction

Idiopathic pulmonary fibrosis (IPF) is a progressive and irreversible interstitial lung disease (ILD) of unknown etiology with a heterogeneous clinical course [1]. Notably, IPF is the most common form of idiopathic interstitial pneumonias with a median survival of 2.5–3.5 years after diagnosis [2]. Recently, pirfenidone, an orally active, small molecule that inhibits synthesis of pro-fibrotic and inflammatory mediators, was approved for the treatment of patients with mild to moderate IPF in the European Union and other countries, as it demonstrated a significant reduction of disease progression and a potential survival benefit [3, 4]. Furthermore, another drug, nintedanib, showed efficacy in the treatment of IPF [5]. However, given the variable course of the disease and the potential adverse effects associated with the substantial costs of these treatments, it is crucial to objectively assess the progression of IPF, in order to identify natural course and treatment effectiveness both in clinical practice and during clinical trials.

Although predictive markers of response to therapy are still missing, pulmonary impairment, symptoms, and serial imaging changes are widely used in monitoring patients. Forced vital capacity (FVC) and diffusing capacity of the lung for carbon monoxide (DLCO) are both recommended in monitoring IPF [1]. However, FVC quantification is sometimes challenging in patients with dyspnea and cough and it might be demanding to differentiate between significant changes that represent disease progression and measurement variability [1, 6]. In addition DLCO is affected by underlying pulmonary vasculopathy and emphysema [1, 7, 8].

In contrast, multidetector computed tomography (MDCT) is a potential alternative to assess “in vivo” the progression of the disease [9]. Despite several MDCT features can be observed in fibrotic interstitial lung disease (reticular pattern, traction bronchiectasis, ground glass opacity, and honeycombing), agreement between readers about the presence and extent of these basic patterns is variable [10–13]. Quantitative computed tomography has the potential advantages of objectivity and ability to identify features that are not visually recognizable. Histogram-based measurements showed moderate correlation with functional impairment and demonstrated significant changes at one-year follow-up, indicating disease progression [14–16]. In chronic obstructive pulmonary disease (COPD), the 15^th^ percentile of lung density histogram is regarded as a useful tool in detecting patients with emphysema [17]. It is therefore hypothesized, that in patients suffering from IPF, specific percentiles of lung density histogram might reflect the extension of MDCT abnormalities and might be considered a biomarker of disease evolution.

The purposes of this study were to describe the changes over time in the extent of parenchymal abnormalities assessed on chest MDCT by both semi-quantitative visual scores (VSs) and automatic histogram-based quantitative analysis and to test the relationship between these two methods of quantification of lung abnormalities in patients suffering from IPF.

## Materials and Methods

### Patients

All procedures were in accordance with the declaration of Helsinki. MDCTs were performed on clinical indication. Informed written consent for examination was obtained from all patients. Clinical records of 256 patients suffering from IPF admitted to our center for interstitial and rare lung disease between August 2006 and December 2013 were reviewed retrospectively. The retrospective analysis was approved by the ethics committee of the medical school of the University of Heidelberg (IRB approval number S-318/2013). All patient records were anonymized and de-identified prior to analysis.

The diagnosis of IPF was confirmed in each patient by a multidisciplinary team discussion meeting (pulmonologists, radiologists, and pathologists experienced in the diagnosis and treatment of IPF) and was based on a review of clinical data, chest MDCT, and histological assessment of lung biopsies. IPF was diagnosed according to the current consensus statement of the American thoracic society/European respiratory society/Japanese respiratory society/Latin American Thoracic Association (ATS/ERS/JRS/ALAT) for patients with first diagnosis after 2011, whereas prior cases were diagnosed following the previous guidelines [1, 18]. Individual therapeutic schemes were selected on the basis of the interdisciplinary statement.

Demographic and clinical data of the study population are shown in Table 1. A total of 40 patients that underwent 2 technically identical MDCT examinations on the same scanner separated by an interval of at least 6 months (median interval between initial and follow-up MDCT: 13 months; interquartile: 10 months; 17 months) were enrolled consecutively. In order to assess whether the range of detectable differences over time in MDCT lung density histogram was measureable, a random population of 20 patients treated with pirfenidone, an anti-fibrotic drug licensed for mild to moderate IPF, and another 20 patients not treated with this drug was composed [19]. The interval time between the initial MDCT and the beginning of the pirfenidone therapy did not exceed about 1/3 of the interval between the initial and the follow-up MDCT scans. Pirfenidone was not clinically indicated for several reasons in the remaining 20 patients [19]. To perform an accurate evaluation in longitudinal changes of lung attenuation histograms, were excluded patients in which initial and follow-up MDCT scans were obtained using different scanner, contrast application (i.e initial contrast-enhanced MDCT and not contrast-enhanced follow-up scan), or reconstructed with different algorithms (i.e filtered back projection vs. iterative) [20]. Details about medical treatment between initial and follow-up MDCTs are shown in Table 1.

**Table 1:** Study population (n = 40) characteristics.

Sex [M/F]	33/7
Initial Age [years]	70 (62–75)
Initial body mass index (BMI) [kg/m2]	27 (24–30)
Relevant comorbidities	
Pulmonary hypertension [number]	3/40
Fibrosis with emphysema^a^ [number]	13/40
Gastro-esophageal reflux disease [number]	6/40
Smoking History	
Never [number]	12/40
Former smokers [number]	25/40
Current smokers [number]	3/40
Pack-years [years]	20 (10;36)
Diagnostic procedures	
Definite usual interstitial pneumonia pattern on MDCT [number]	21/40
Surgical biopsy [number]	19/40
Interval IPF diagnosis and 1st MDCT [months]	13 (0–35)
Initial pulmonary function tests	
VC [% predicted]^b^	71 (58;84)
TLC [% predicted]^b^	61 (58;82)
DLCO [% predicted]^c^	40 (28;57)
PaO_2_ [mmHg]	66 (63;72)
Initial O_2_ therapy [number]	6/40
Therapy prior initial MDCT	
N-acetylcysteine only [number]	27/40
Steroids and N-acetylcysteine [number]	8/40
Combined immunosuppressive therapy (N-acetylcysteine, steroids, and immunomodulator^d^) [number]	5/40
Therapy between initial and follow-up MDCT	
N-acetylcysteine only [number]	5/40
Steroids and N-acetylcysteine [number]	2/40
Combined immunosuppressive therapy (N-acetylcysteine, steroids, and immunomodulator^d^) [number]	13/40
Pirfenidone alone [number]	13/40
Pirfenidone and N-acetylcysteine [number]	1/40
Pirfenidone, N-acetylcysteine, and steroids [number]	3/40
Pirfenidone and steroids [number]	3/40
Sildenafil	1/40
Bosentan	1/40
Initial and follow-up MDCT interval [months]	13 (10–17)

Summary of gender, age, body mass index (BMI), smoking history, comorbidities, diagnostic procedures, pulmonary function tests [including vital capacity (VC), total lung capacity (TLC), diffusing capacity of the lung for carbon monoxide (DLCO), and arterial pressure of oxygen (PaO_2_)], therapy prior and after initial multidetector computed tomography (MDCT), and interval between initial and follow-up MDCT data of the idiopathic pulmonary fibrosis (IPF) patients that constituted the study population. Data are presented as absolute number of patients (n) or median and interquartile range (in brackets).

^a^Was considered the presence or absence of emphysema additionally to fibrosis.

^b^One value is missing due to patient inability to perform pulmonary function tests.

^c^Seven values are missing due to patient inability to perform the DLCO test.

^d^Immunomodulator indicates azathioprine and\or cyclophosphamide at a dosage clinically indicated.

### Multidetector Computed Tomography

In all IPF patients, thin-section MDCT of the entire chest was routinely performed at inspiratory breath-hold in supine position using spiral mode scanning. Prior to the examination, all patients were trained to achieve a full end-inspiratory breath-hold after automatic patient instruction. Twenty-nine patients underwent the pair of baseline and follow-up MDCT scans on a 4-slice scanner (Volume Zoom, Siemens AG, Forchheim, Germany) and 11 patients on a 64-slice scanner (Somatom Definition AS, Siemens AG, Forchheim, Germany). The technical parameters for the 4-slice scanner were as follows: tube voltage 120 kV, effective tube current-time product of 70 mAs, collimation of 4x1.25 mm, rotation time 0.5 sec, and pitch of 2 (typically DLPw = 127 mGy cm, E = 1.6 mSv, and scan time = 18 sec). The acquisition parameters for the 64-slice scanner were as follows: tube voltage 120 kV, median effective tube current-time product of 67 mAs (range 48–151), collimation of 64x0.6 mm, rotation time of 0.33 sec, and pitch of 1.5 (typically DLPw = 37 mGy cm, E = 0.6 mSv, and scan time = 3 sec). Two patients were scanned both at baseline examination and at follow-up with contrast enhanced MDCT on the 4-slice scanner with identical administration (120ml Iopamidol-300, Bracco Imaging, Konstanz, Germany @ 3.5ml/s). Baseline and follow-up scans were started automatically by bolus triggering with a region-of-interest (ROI) located in the pulmonary trunk, and scanning protocol as mentioned above.

Image reconstruction was performed with a slice thickness of 1.25 and 1 mm increment. Since medium-soft kernels are more accurate and less noisy than sharp kernels for computer based quantitative analysis, either B40f kernel (4-slice scanner) or iterative I40f algorithm (64-slice scanner) were used [15]. The scale of attenuation coefficients with these systems ranged from -1024 HU to +3071 HU. The systems were calibrated with a standard phantom for water in quarterly period and after major maintenance, as well as for air daily. MDCT scans affected by severe respiratory artifacts were not encountered in this study.

### Visual scoring and automatic evaluation at MDCT

MDCT images for each patient were reviewed by a trainee (D.C.) with 2 year of experience in thoracic imaging, who was blinded to individual therapy, clinical findings and histological data, after 6 months of focused training for ILD under interactive guidance of experienced chest radiologists (C.P.H and J.D.). For each patient, all the MDCT scans were scored on a certified PACS workstation simultaneously, using a window setting for lung parenchyma (center, -600 HU; width, 1600 HU).

MDCT scans were visually evaluated at six axial levels selected by the reader as follows: 1) the aortic arch, 2) the carina, 3) the pulmonary venous confluence, 4) the midpoint between level 3 and level 5, 5) 1 cm above the dome of the right hemi-diaphragm and 6) 2 cm below the dome of the right hemi-diaphragm [21]. MDCT findings were interpreted on the basis of the recommendations suggested by the Fleischner Society nomenclature committee [10]. The reader quantified semi-quantitatively in each slice the overall extent of interstitial lung disease (OE), defined by the sum of ground glass opacities (GGO), reticular opacities (RET), honeycombing (HC), and consolidations (CONS). Afterwards the reader quantified in each slice the individual amount of GGO, RET, HC, CONS and emphysema (EMP). Each score was calculated as the percentage of lung parenchyma involved to the nearest 5%. In each patient, the total extent of OE, GGO, RET, HC, CONS and EMP were derived by averaging the scores obtained at the six axial levels described above.

**Fig 1:**
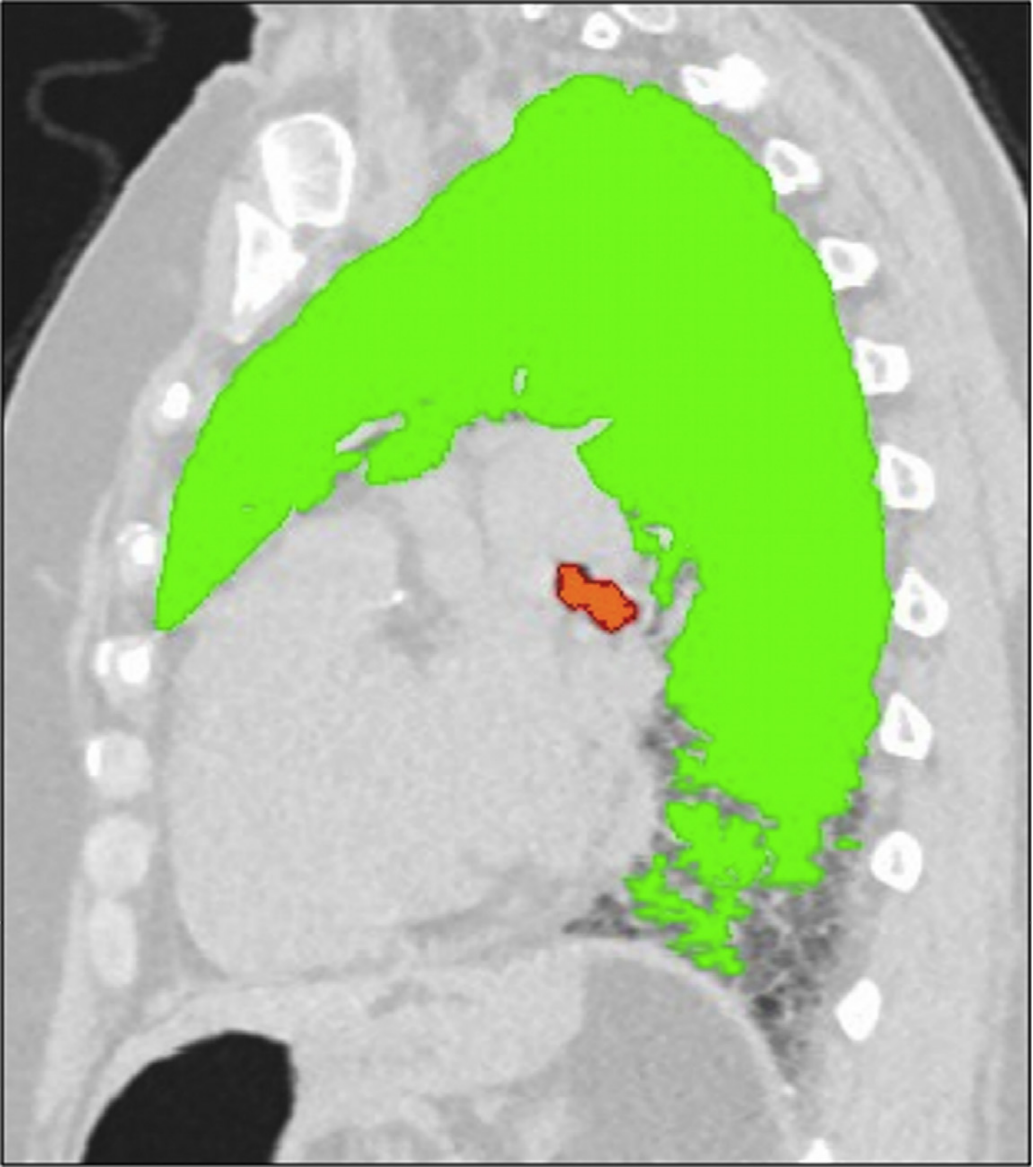
Visualization of the fully automatic lung parenchyma segmentation as obtained by in-house YACTA software. Sagittal reconstruction image of a non-enhanced MDCT scan obtained from a patient suffering from idiopathic pulmonary fibrosis (IPF) not included in the current trial. YACTA software automatically segmented lung parenchyma and trachea-bronchial tree, emphasized as green and orange overlay respectively (window width: 1600 HU; level: -600 HU). Note that the segmentation algorithm fails to segment portions of the lung parenchyma in the sub-pleural space of the recessus, due to its similar density to the chest wall. (MDCT = multidetector computed tomography).

The lung density histogram of each CT was obtained using the in-house YACTA software, which was primarily developed for the emphysema quantification [22]. The stack of around 300 DICOM images per patient was analyzed fully automatically in an unattended mode. Soft tissues (> -750 HU), lungs (< -500 HU), and tracheobronchial tree were found fully automatically using both threshold values and anatomical knowledge-based algorithms without manual interaction (Fig 1). In each MDCT the reader assessed the quality of the volume automatically segmented. The HU value of the range 10^th^-90^th^ percentile in 5^th^ percentiles steps was derived from the histogram recording the densities of all lung voxels for each MDCT.

### Pulmonary function tests

Body plethysmography (MasterScreen Body; E. Jaeger, Hoechberg, Germany) was performed according to the guidelines of the ERS and the standards of the ATS [23]. Pulmonary function tests (PFTs) were obtained within 30 days of MDCT scans. The European Coal and Steel Community—predicted values were selected as our in-house standard and reflect the individually measured value in relation to an age- and height-matched control population given in percentage [24]. The “%” symbol following the PFT parameters indicates the predicted values in percent. The changes in both vital capacity (VC%) and diffusing capacity of the lung for carbon monoxide (DLCO%) between the initial and the follow-up PFTs were used for the correlation analysis [1].

### Data evaluation and statistical analysis

Individual longitudinal changes per year in VSs (Δ VSs %/y), in every 5^th^ percentile included in the range of 10^th^-90^th^ percentile of lung density histogram (Δ PERC HU/y), and in PFTs (Δ PFTs %/y) were computed as the difference between the initial and follow-up MDCTs results divided by the interval. In order to identify a measurable difference in MDCT density, the percentiles of the attenuation histogram characterized by the largest difference and the lowest overlap in longitudinal HU changes between treated and untreated patients were identified. Pearson correlation analysis was used to evaluate the relationship between VSs, selected PERC, and PFTs%. All data were recorded using a dedicated database (Excel 2010, Microsoft Corp., Redmond, WA) and analyzed using SPSS version 20 (SPSS, Inc., Chicago, IL). A *p* value less than 0.05 was considered significant.

## Results

### Visual scoring and automatic evaluation at MDCT

Table 2 summarizes both baseline and sequential VSs changes per year (Δ VSs). At follow-up MDCT, the OE increased in median of 5%/y (interquartile: 0%/y; +11%/y). Regarding individual pattern, RET and HC extent showed a median increment of 2%/y (interquartile: -1%/y; +5%/y) and 1%/y (interquartile: 0%/y; +2%/y) respectively; in addition GGO (median: 0%/y; interquartile: -2%/y; +3%/y), and CONS (median: 0%/y; interquartile: 0%/y; 0%/y) extents were stable., Twenty-seven patients showed no signs of emphysema at baseline. Thirteen patients manifested emphysema at MDCT regardless the percentage of parenchyma involved. Excluding 2/13 patients with an emphysema visual score of 18% and 24%, the remaining 11/13 patients manifested a maximum amount of emphysema of 8%.

**Table 2:** Visual scores obtained at multidetector computed tomography (MDCT).

Initial MDCT	
overall extent [%]	32 (23;51)
ground-glass opacities [%]	6 (2;15)
reticular opacities [%]	20 (14;29)
honeycombing [%]	1 (0;3)
consolidations [%]	0 (0;0)
emphysema [%]	0 (0;3)
Longitudinal changes at 1-year follow-up (Δ)	
Δ overall extent [%/y]	+5 (0;+11)
Δ ground-glass opacities [%/y]	0 (-2;+3)
Δ reticular opacities [%/y]	+2 (-1;+5)
Δ honeycombing [%/y]	+1 (0;+2)
Δ consolidations [%/y]	0 (0; 0)
Δ emphysema [%/y]	0 (0;0)

Summary of the visual scores, expressed as percent of parenchyma involved (%), obtained at initial multidetector computed tomography (MDCT) and their longitudinal changes at 1-year (Δ), expressed as percent per year (%/y). Ground-glass opacities, reticular opacities, honeycombing, consolidations, and emphysema were defined according to the recommendations suggested by the Fleischner Society nomenclature committee [10]. Data are expressed as median and interquartile ranges (in brackets).

**Fig 2:**
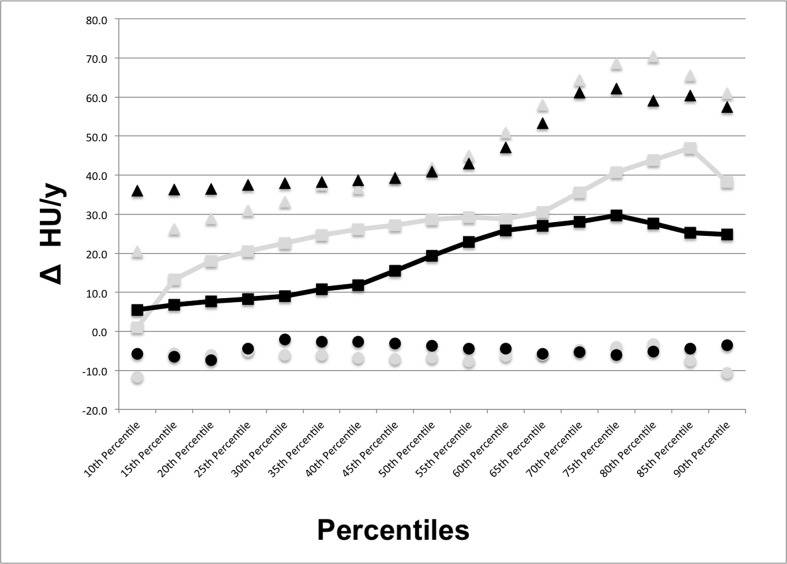
Median density changes at 1-year follow-up in the range of 10^th^-90^th^ percentile of the MDCT attenuation histogram for patients suffering from idiopathic pulmonary fibrosis (IPF) treated and untreated with pirfenidone. The largest difference with the lowest overlap between treated and untreated patients in longitudinal HU changes of the attenuation histogram was detected in the 40th and in the 80th percentiles. Black squares = median increase of each 5^th^ percentile step included in the 10^th^-90^th^ range of the lung density histogram for patients treated with pirfenidone; black triangles and circles = interquartile range of the increase in each 5^th^ percentile step included in the 10^th^-90^th^ range of the lung density histogram for patients treated with pirfenidone; grey squares = median increase of each 5^th^ percentile step included in the 10^th^-90^th^ range of the lung density histogram for patients not treated with pirfenidone; grey triangles and circles = interquartile range of the increase in each 5^th^ percentile step included in the 10^th^-90^th^ range of the lung density histogram for patients not treated with pirfenidone. (Δ HU/y = changes in Hounsfield units per year; MDCT = multidetector computed tomography).

**Fig 3:**
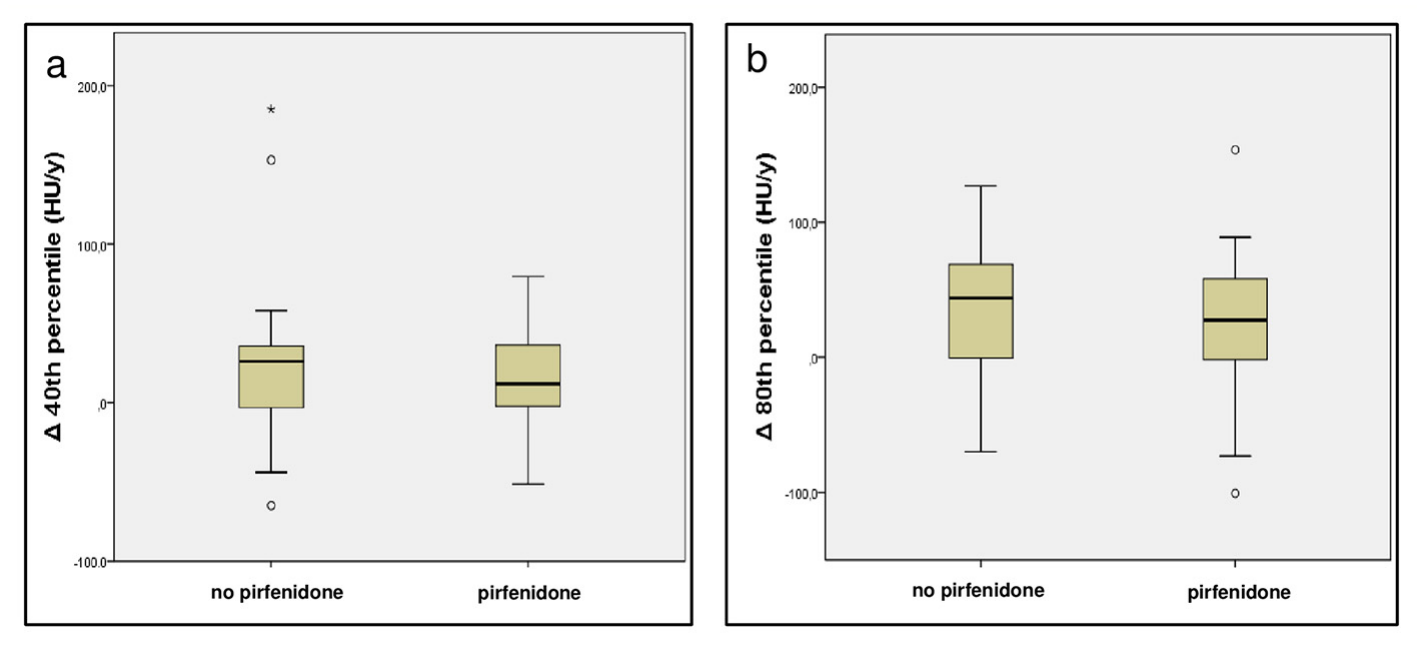
Distribution of the density changes at 1-year in the selected percentiles from the MDCT attenuation histogram of patients suffering from idiopathic pulmonary fibrosis (IPF) treated and untreated with pirfenidone. Box and Whisker plots represent the 40^th^ percentile (a) and the 80^th^ percentile distributions (b) of lung density histogram as observed in patients treated with pirfenidone and in patients not treated with pirfenidone. The central line represents the median, the yellow box encompasses the 25^th^-75^th^ percentiles, whiskers show the 10^th^-90^th^ percentile, and the empty circles represent individual outliers. (HU/y = Hounsfield units per year; Δ 40^th^ percentile = changes at 1-year in the 40^th^ percentile of the attenuation histogram; Δ 80^th^ percentile = changes at 1-year in the 80^th^ percentile of the attenuation histogram; MDCT = multidetector computed tomography).

For each MDCT the volume automatically segmented was considered satisfactory by the reader. Fig 2 shows the median HU increase over 1-year in each 5^th^ percentile included in the range 10^th^-90^th^ percentile of lung density histogram for both treated and untreated patients. The largest difference with the lowest overlap between treated and untreated patients in longitudinal HU changes of the attenuation histogram was detected in the 40^th^ and in the 80^th^ percentiles. The initial median density of the 40^th^ percentile was -822 HU (interquartile: -861 HU; -777 HU), while the 80^th^ percentile showed a median density of -595 HU (interquartile: -674 HU; -518 HU); at follow-up MDCT, the 40^th^ percentile demonstrated a median increase of 22 HU (interquartile: -3 HU; +37 HU) and the density of the 80^th^ percentile increased of 35 HU (interquartile: -3 HU; +65 HU). In particular, the median HU change of the 40^th^ percentile was of 12 HU (interquartile: -3 HU; +39 HU) in patients with the anti-fibrotic treatment and equal to 26 HU (interquartile: -7 HU; +37 HU) in patients without specific anti-fibrotic therapy (Fig 3a). In the 80^th^ percentile (Fig 3b), patients treated with pirfenidone showed a median density increment of 28 HU (interquartile: -5 HU; +59 HU) whereas the median density increase in patients without specific anti-fibrotic therapy was 44 HU (range: -3 HU; +70 HU).

### Pulmonary function tests

The median VC% impairment was 4%/y (interquartile: -10%/y; +2%/y), while the reduction of DLCO% was 2%/y (range: -13%/y; +2%/y). Due to worsening of the respiratory symptoms, the follow-up VC% was not obtained in 2/40 patients, whereas follow-up DLCO% measurement was not performed in 7/40 patients for the same reason.

### Correlation analysis

**Fig 4:**
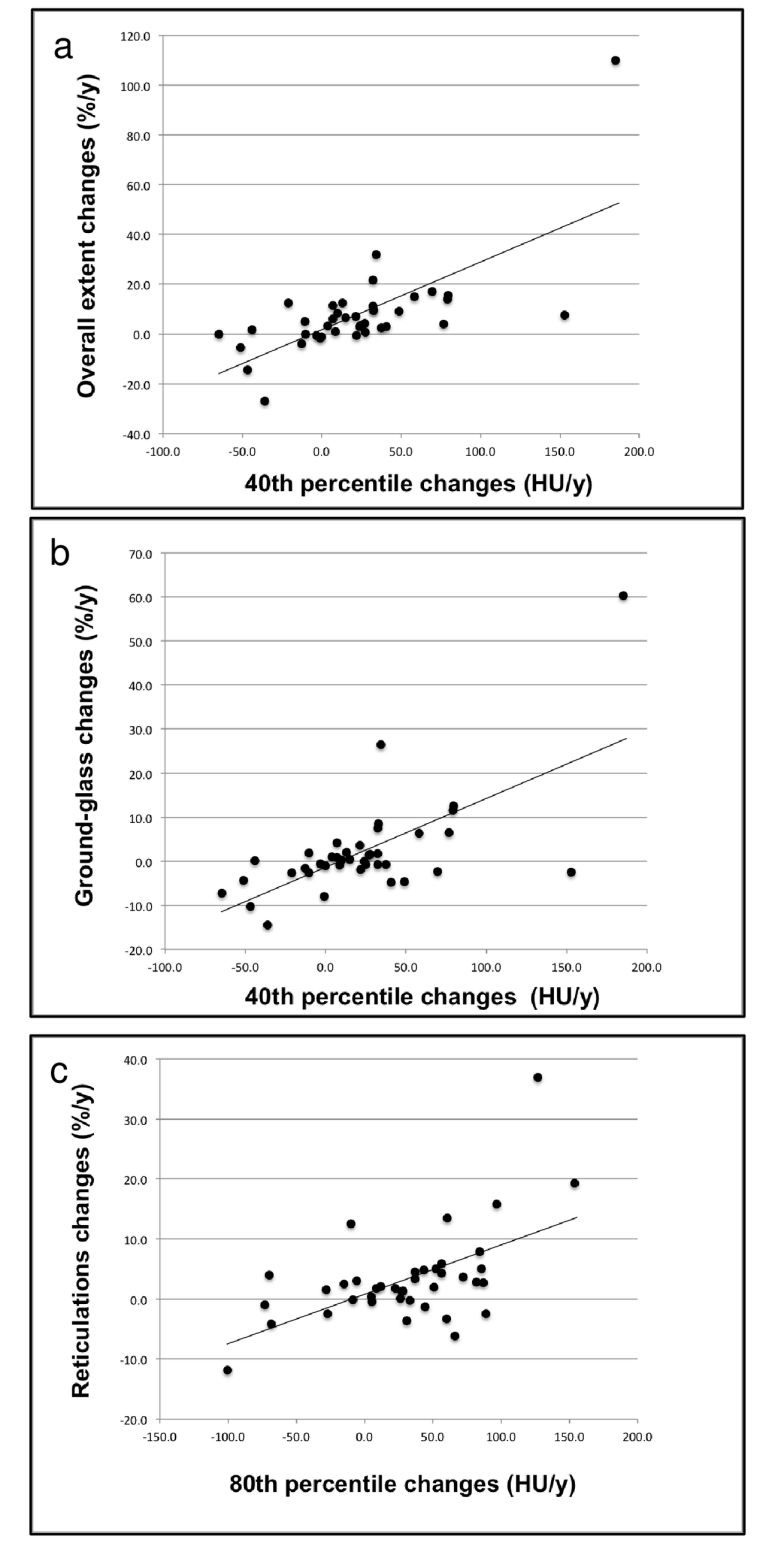
Correlations between changes at 1-year in selected percentiles of MDCT attenuation histogram and visual scores. Dot plots with linear regression curves for changes in overall extent of parenchymal abnormalities plotted against density variation in the 40^th^ percentile of the MDCT attenuation histogram (r = 0.69, *p* < 0.001) (a), for the changes in ground-glass opacity extent plotted against density variation in the 40^th^ percentile of the MDCT attenuation histogram (r = 0.66, *p* < 0.001) (b), and for changes in reticulations extent plotted against density variation in 80^th^ percentile of the MDCT attenuation histogram (r = 0.56, *p* < 0.001) (c). (%/y = percent per year; HU/y = Hounsfield units per year; MDCT = multidetector computed tomography).

**Fig 5:**
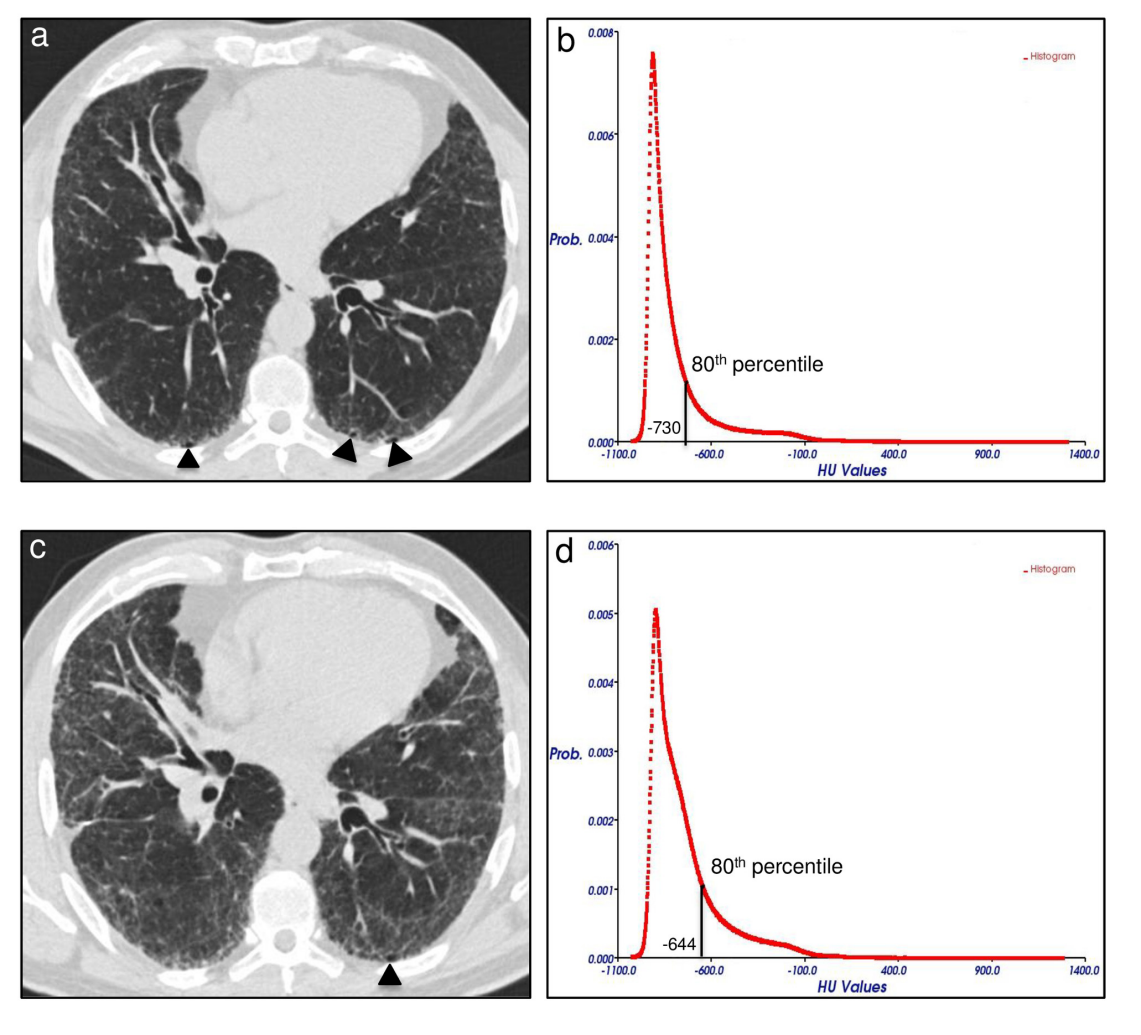
Variation of disease extent at follow-up MDCT in a 53 years-old man suffering from idiopathic pulmonary fibrosis (IPF) not treated with pirfenidone. Axial MDCT image at level 5 (1 cm above the right hemi-diaphragm as described in the text) of the initial examination shows mild reticular opacities and minimal honeycombing (arrowheads) in a sub-pleural distribution (a). The 80^th^ percentile of the initial MDCT attenuation histogram corresponded to -730 HU (b). Follow-up MDCT scan at the same axial level after 17 months shows a predominant increase of reticulations (visual score = +22%) and unmodified honeycombing extent (arrowhead) (c). An increase of 86 HU in the density of the 80^th^ percentile was seen between initial and follow-up MDCT attenuation histograms (d). (Prob. = probability; HU = Hounsfield units; MDCT = multidetector computed tomography).

The Pearson correlation coefficient between the selected percentiles of lung density histogram and VSs are given in Table 3. The Δ OE showed a greater correlation with the Δ 40^th^ PERC (*r* = 0.69, *p* < 0.001; Fig 4a), as compared to the Δ 80^th^ PERC (*r* = 0.58, *p* < 0.001). Additionally, the Δ 40^th^ PERC correlated more tightly with the Δ GGO (*r* = 0.66, *p* < 0.001; Fig 4b) as compared to the remaining abnormalities. Conversely, the Δ 80^th^ PERC demonstrated higher correlation with the Δ RET (*r* = 0.56, *p* < 0.001; Figs 4c and 5) relative to the other patterns. The Δ HC demonstrated a significant correlation with the Δ 40^th^ PERC (*r* = 0.48, *p* = 0.002), whereas no significant correlation was observed between the same pattern and the variation of lung density in the 80^th^ percentile.

**Table 3:** Correlation analysis between selected percentiles of multidetector computed tomography (MDCT) attenuation histogram and visual scores (VSs).

Initial MDCT				
VSs	40th percentile [HU]	*p* value	80th percentile [HU]	*p* value
OE [%]	0.79	**<0.001**	0.75	**<0.001**
GGO [%]	0.52	**0.001**	0.32	**0.002**
RET [%]	0.64	**<0.001**	0.67	**<0.001**
HC [%]	0.16	0.312	0.26	0.091
Longitudinal changes at 1-year follow-up (Δ)				
Δ VSs	Δ 40^th^ percentile [HU/y]	*p* value	Δ 80th percentile [HU/y]	*p* value
Δ OE [%/y]	0.69	**<0.001**	0.58	**<0.001**
Δ GGO [%/y]	0.66	**<0.001**	0.47	**0.002**
Δ RET [%/y]	0.43	**0.003**	0.56	**<0.001**
Δ HC [%/y]	0.48	**0.002**	0.27	0.091

Summary of correlation analysis between visual scores (VSs) and selected percentiles obtained from multidetector computed tomography (MDCT) attenuation histogram at initial MDCT and at 1-year follow-up. Pearson r coefficients were calculated for overall extent of abnormalities (OE), ground-glass opacities extent (GGO), reticulations extent (RET), and honeycombing extent (HC), expressed as percent of parenchyma involved (%), with the density, expressed as Hounsfield Unit (HU), of both the 40^th^ and the 80^th^ percentiles obtained from the MDCT attenuation histogram at baseline. The same coefficients were obtained for longitudinal changes at 1-year (Δ) in VSs, expressed as percent per year (%/y), with the variation in density, expressed as Hounsfield Unit per year (HU/y), of both the 40^th^ and the 80^th^ percentiles obtained from the MDCT attenuation histogram. In bold *p* values statistically significant (*p*<0.05).

The correlation coefficients obtained between the selected percentiles of lung density histogram, VSs, and PFT% are listed in Table 4. The Δ VC% showed a negative, good correlation with Δ OE (*r* = -0.63, *p* < 0.001). However, both the Δ GGO (*r* = -0.36, *p* = 0.031) and Δ RET (*r* = -0.43, *p* = 0.005) extent demonstrated a significant but weak negative relationship with the Δ VC%. Changes in HC extent was not significantly correlated with Δ VC%. The Δ VC% demonstrated a similar negative correlation with both the Δ 40^th^ PERC (*r* = -0.58, *p* < 0.001) and the Δ 80^th^ PERC (*r* = -0.57, *p* < 0.001). The Δ VSs and Δ DLCO% were not significantly correlated, while was found a significant, although weak, relationship between Δ DLCO % and Δ 40^th^ PERC (*r* = -0.48, *p* = 0.008) as well as with Δ 80^th^ PERC (*r* = -0.40, *p* = 0.028).

**Table 4:** Correlation analysis of pulmonary function tests (PFTs) with visual scores (VSs) and selected percentiles obtained from multidetector computed tomography (MDCT) attenuation histogram.

VSs, PERC	VC [%]	*p* value	DLCO [%]	*p* value
OE [%]	-0.57	**<0.001**	-0.49	**0.004**
GGO [%]	-0.45	**0.004**	-0.36	**0.038**
RET [%]	-0.31	0.053	-0.23	0.207
HC [%]	-0.05	0.754	-0.56	**0.001**
40^th^ percentile [HU]	-0.51	**0.001**	-0.50	**0.003**
80^th^ percentile [HU]	-0.56	**<0.001**	-0.62	**<0.001**
Δ VSs, Δ PERC	Δ VC [%/y]	*p* value	Δ DLCO [%/y]	*p* value
Δ OE [%/y]	-0.63	**<0.001**	-0.16	0.395
Δ GGO [%/y]	-0.36	**0.031**	-0.04	0.839
Δ RET [%/y]	-0.43	**0.005**	-0.25	0.189
Δ HC [%/y]	-0.23	0.156	+0.05	0.783
Δ 40^th^ percentile [HU/y]	-0.58	**<0.001**	-0.48	**0.008**
Δ 80^th^ percentile [HU/y]	-0.57	**<0.001**	-0.40	**0.028**

Summary of correlation analysis between pulmonary function tests (PFTs), visual scores (VSs), and selected percentiles (PERC) obtained from multidetector computed tomography (MDCT) attenuation histogram at initial MDCT and at 1-year follow-up. Pearson r coefficients were calculated for overall extent of abnormalities (OE), ground-glass opacities extent (GGO), reticulations extent (RET), honeycombing extent (HC) [all expressed as percentage of parenchyma involved (%)], and the density, expressed as Hounsfield Unit (HU), of selected percentiles obtained from the MDCT attenuation histogram with the percent predicted (%) of vital capacity (VC) and of diffusing capacity of the lung for carbon monoxide (DLCO) at baseline. The same coefficients were obtained for both longitudinal changes at 1-year (Δ) in VSs, expressed as percent per year (%/y), and in the density, expressed as Hounsfield Unit per year (HU/y), of selected percentiles obtained from the MDCT attenuation histogram with the variation, expressed as percent predicted per year (%/y), of VC% and of DLCO%. In bold *p* values statistically significant (*p*<0.05).

## Discussion

This study describes the intra-individual changes over 1 year in a visual and a fully automatic histogram-based quantitative evaluation of MDCT in patients suffering from IPF. The visual assessment of the follow-up MDCT demonstrates an increase in the overall extent of the disease with a predominant increment of the fibrotic abnormalities. Changes in the density values of the 40^th^ and the 80^th^ percentile of the MDCT attenuation frequency histogram were identified as promising parameters for monitoring the disease extent. The correlation analysis between changes after 1 year in VSs and in selected percentiles suggested that the 40^th^ percentile of lung density histogram, representing the low-density parenchymal areas (i.e. 40% of all lung voxels are of lower density while 60% are of higher density), might reflect the overall extension of the abnormalities, including the GGO, which hypothetically represents potentially reversible acute inflammation [25, 26]. Additionally, the 80^th^ percentile of attenuation histogram, representing the high-density parenchymal areas (i.e. 80% of all lung voxels are of lower density while 20% are of higher density), might reveal the progression of the reticular opacities.

Since visual assessment is time-consuming, suffers from low reproducibility and even intra-individual variation, it is consecutively not used in clinical routine [11–13]. The role of quantitative MDCT indexes of interstitial lung diseases is challenging in this high-contrast organ, consisting of low and high-density compartments [16]. In patients suffering from IPF, the histogram-based approach was used more extensively and showed a reasonable correlation with the pulmonary function tests [14]. In a previous study, skewness and kurtosis of MDCT attenuation histograms were used as quantitative indexes and their changes over time suggested that these parameters might be used to identify progression of fibrosis [16]. However, skewness and kurtosis are not “user friendly” in the clinical routine [27]; additionally, in a recent study of Kim et al., longitudinal changes in kurtosis were not associated with FVC and DLCO impairment [28]. Therefore, in the present study, a novel and easier approach was introduced for the automatic estimation of disease progression, based on density changes of selected percentiles derived from the MDCT lung density histogram. The lung density increase in the 80^th^ percentile of frequency histogram was of about 30–40 HU, in contrast to an increment of 10–20 HU detected in the 40^th^ percentile. Especially changes in the 80^th^ percentile are of a significant amount, which is crucial in a high-contrast organ such as the lung, whose density is also influenced by different respiratory levels. In the majority of cases, the MDCT abnormalities detected in IPF patients consisted of mixed ground-glass opacities, reticulations and honeycombing that resulted in a wide range of attenuation values for the respective pattern. For instance, an increase of the gross cystic changes results in a decrease of the average regional HU values, possibly due to the air inside the cysts, in contrast to an increment of reticular opacities and ground-glass opacities extents that causes an increase of the MDCT attenuation [29]. For this reason, the increase of the 80^th^ percentile lung density, which represented the reticular opacities progression, was higher as compared to the increment observed in the 40^th^ percentile, which probably reflected a combination of a predominant ground-glass pattern mixed with reticular opacities and honeycombing. Similarly to the findings obtained by Yoon et al., in this study a relatively good correlation was demonstrated between the automatic and the visual quantification of the lung abnormalities extent, though higher correlation was seen between the visual score of reticulations and correspondent automatic quantification (Table 3) [13]. Furthermore, in another series the association between the visual and the automatic quantification of fibrotic pattern was weak [9]. For these reasons, the automatic quantification based on density changes of selected percentiles derived from attenuation histogram might be more reliable in distinguishing the different IPF abnormalities than Gaussian histogram normalized correlation (GNHC) and texture analysis based on statistical descriptors of frequency histogram (i.e mean, standard deviation, skewness and kurtosis) used in the above mentioned studies [9, 13, 30].

The assessment of the relationship between MDCT features and functional status is challenging. MDCT is unable to quantify the absolute severity of inflammatory and fibrotic disease [29]. However, the correlation with both the VC% and the DLCO% decline achieved by changes of histogram attenuation in the selected percentiles were higher than the relationship observed in previous studies between statistical descriptors of lung density histogram (mean, standard deviation, skewness and kurtosis) and the functional impairment [13–15]. These results confirmed that histogram analysis based on the selected percentiles do better represent the decline of lung function over time than the investigation of the other frequency histogram features above mentioned.

There are several limitations in this study besides those already described above. First, it is a retrospective data analysis on a small number of patients from a single center. However, the presence of patients from only one center reduced the inter-scanner variability, which is a well-known drawback and limitation of any quantitative analysis and especially the automatic evaluation [14]. Second, no spirometric gating in the acquisition of chest MDCT images was used, but each patient was trained to achieve a full end-inspiratory breath-hold. Also, Best et al. suggested that spirometric standardization might not be necessary for routine use [16]. Third, visual scores at CT were obtained by a single not-experienced reader. However, it is well-known that visual assessment suffers from inter-individual and even intra-individual variation, thus the visual analysis was not based upon a multi-reader special-expert board, but upon a constant focus trained single reader under interactive guidance of experienced chest radiologists [11–13]. In addition, correlations with percentiles obtained in the present study were good, therefore they are expected higher if visual scores are obtained by experienced readers. Fourth, there is a weakness in the automatic segmentation provided by the in-house software, which was initially developed for emphysema quantification with corresponding algorithms for lung contour detection. The software expected a low-density lung parenchyma while IPF-patients show a high-density lung parenchyma, leading to possible failures in the segmentation of the sub-pleural regions. Since IPF manifests to a relevant amount in the sub-pleural space, the segmentation algorithm fails to include parts of the affected lung as seen in a patient not included in this trial shown in Fig 1. This requires more sophisticated segmentation algorithms that take anatomical knowledge (rib, liver, etc.) into account and\or allow manual correction of the segmented volume. Such tools are under development but were not implemented in the YACTA software at the study time. This might have caused an underrepresentation of the high-density parts of the disease leading to higher values of e.g. the 80^th^ percentile. However, during the planning phase of a software manual correction tool, was found a difference of 70 ml (around 1–2% of the total lung volume) between the manual and the fully automatic segmentation volume. This difference might be regarded marginal considering that might be nearly constant in the longitudinal MDCTs during the course of the disease. Fifth, the CT measurements were not corrected for eventually different lung volumes. This would affect density measures significantly. Neither the visual scores, nor the attenuation percentiles were normalized for lung volumes. This should not affect the correlations results obtained in the present study between such methods of quantification. However, for future studies lung volume normalization is required, in order to eliminate the well-known limitation of sub-optimal inspiration during MDCT scan [31]. Sixth, the patients analyzed were treated or untreated resulting in very small subgroups. Since the purpose of this analysis was not to show treatment success but only whether a follow-up can be quantified automatically, the included population was chosen as it happens nowadays in clinical routine. This includes treated and untreated patients with the aim of keeping the CT technology constant. Seventh, our study population included two patients who underwent contrast-enhanced MDCT scans in order to mirror the typical clinical environment. Iodinated contrast medium affects the lung attenuation [32]. However, to reduce this limitation, both baseline and follow-up MDCTs scan were obtained with identical bolus triggering technique on the same scanner. Lastly, MDCT lung attenuation could be affected by congestive hearth failure and pulmonary embolism, which determine significant alterations of the lung density histogram, such as a shift to higher HU values or a reduction of skewness and kurtosis [33, 34]. However, in the present study, the patients were regularly screened by echocardiography and hematic levels of brain natriuretic peptide (BNP). None of them suffered from congestive heart failure or pulmonary embolism at MDCT time.

## Conclusions

Visual quantification of parenchymal abnormalities at multidetector computed tomography over 1 year in IPF patients showed an increase in the overall extent of the disease, with a predominant increment of the fibrotic abnormalities. The density changes in the 40^th^ percentile might reflect the overall extension of the lung abnormalities, in particular of the ground-glass pattern, which could represent potentially reversible acute inflammation; in addition, the change in the 80^th^ percentile might reveal the progression of the irreversible fibrotic reticular opacities. Future studies with a larger number of patients might focus upon these values.
